# Supplementary material for: Deep Learning Approach for Predicting the Therapeutic Usages of Unani Formulas towards Finding Essential Compounds
Source: Life (Basel). 2023 Feb 3;13(2):439. doi: 10.3390/life13020439 (PMC9959740; doi:10.3390/life13020439)
Supplement: Supplementary file 1 [file life-13-00439-s001.zip › life-2139547-supplementary.docx]

**Supplementary Table S1.** List of important metabolites for each disease class extracted from best prediction model using variable importance of Deep Neural Network.

| **No** | **Feature ID** | **Metabolites** | **Weight** |
| --- | --- | --- | --- |
| Class 3 - The Digestive System | | | |
| 1 | 4297 | bis(trimethylsilyl) octanedioate | 0.5150 |
| 2 | 2546 | (2S,3S,4S,5R,6R)-6-[[(3S,4R,4aR,6aR,6bS,8S,8aR,9R,10R,12aS,14aR,14bR)-8,9-dihydroxy-4-(hydroxymethyl)-4,6a,6b,11,11,14b-hexamethyl-10-[(E)-2-methylbut-2-enoyl]oxy-8a-[[(E)-2-methylbut-2-enoyl]oxymethyl]-1,2,3,4a,5,6,7,8,9,10,12,12a,14,14a-tetradecahydropicen-3-yl]oxy]-3,4,5-trihydroxyoxane-2-carboxylic acid | 0.3950 |
| 3 | 3450 | 6H-dibenzo[b,d]pyran-6-one | 0.2980 |
| 4 | 2018 | trimethylsilyl 4-trimethylsilyloxybutanoate | 0.2450 |
| 5 | 2809 | lyratol C | 0.2450 |
| 6 | 3057 | trimethylsilyl 3-phenyl-2-trimethylsilyloxyiminopropanoate | 0.2430 |
| 7 | 3813 | epithienamycin E | 0.2190 |
| 8 | 3122 | bis(trimethylsilyl) 2-oxopentanedioate | 0.2140 |
| 9 | 2356 | 9(S)-HOTrE | 0.2030 |
| 10 | 999 | Dalbergioidin | 0.1970 |
| 11 | 1557 | cimifoetiside A | 0.1950 |
| 12 | 987 | 5-Methoxy-N,N-dimethyl-tryptamine Nb-oxide | 0.1910 |
| 13 | 1835 | (2S,3S,4S,5R,6R)-6-[[(3S,4R,4aR,6aR,6bS,8S,8aR,9R,10R,12aS,14aR,14bR)-8a-(acetyloxymethyl)-8,9-dihydroxy-4-(hydroxymethyl)-4,6a,6b,11,11,14b-hexamethyl-10-[(E)-2-methylbut-2-enoyl]oxy-1,2,3,4a,5,6,7,8,9,10,12,12a,14,14a-tetradecahydropicen-3-yl]oxy]-3,5-dihydroxy-4-[(2S,3R,4S,5S,6R)-3,4,5-trihydroxy-6-(hydroxymethyl)oxan-2-yl]oxyoxane-2-carboxylic acid | 0.1750 |
| 14 | 3045 | quercetin 7,4'-di-O-β-D-glucoside | 0.1730 |
| 15 | 1836 | Phenethylamine | 0.1470 |
| Class 6 - Female-Specific Diseases | | | |
| 1 | 2739 | D-myo-inositol 1,2,5,6-tetrakisphosphate | 0.3400 |
| 2 | 3550 | (R)-4''-methoxydalbergione | 0.2740 |
| 3 | 2151 | Kaempferol 3-(6G-malonylneohesperidoside) | 0.2350 |
| 4 | 582 | butin | 0.1550 |
| 5 | 3286 | Manghaslin | 0.1360 |
| 6 | 1041 | Delphin | 0.1190 |
| 7 | 3491 | Myricetin 3-(2G-rhamnosylrutinoside) | 0.0959 |
| 8 | 484 | Petunidin 3-O-beta-D-glucopyranoside | 0.0655 |
| 9 | 3559 | Delphinidin 3-(6''-malonylglucoside) | 0.0517 |
| 10 | 640 | dalcochinin-8'-O-β-D-glucoside | 0.0491 |
| 11 | 2603 | Malvidin | 0.0277 |
| 12 | 2634 | (R)-4-hydroxy-1-methyl-L-proline | 0.0155 |
| 13 | 1353 | Delphinidin 3-(6-malonylglucoside)-3',5'-di-(6-p-coumaroylglucoside) | 0.0110 |
| Class 8 - The Heart and Blood Vessels | | | |
| 1 | 2311 | kaempferol 3-O-[α-L-rhamnopyranosyl(1→2)-β-D-galactopyranosyl]-7-O-α-L-rhamnopyranoside | 0.5910 |
| 2 | 626 | Succinic acid | 0.5160 |
| 3 | 3460 | Kinocoumarin | 0.0594 |
| 4 | 902 | Betnovateat | 0.0489 |
| 5 | 40 | Linaloyl acetate | 0.0367 |
| 6 | 2949 | BETAMETHASONE VALERATE | 0.0167 |
| 7 | 2002 | 3,5,6-Trihydroxy-7,4'-dimethoxyflavone | 0.0119 |
| Class 10 - Male-Specific Diseases | | | |
| 1 | 1639 | oleuropein | 0.5350 |
| 2 | 333 | Obtusifoliol | 0.5230 |
| 3 | 1362 | Methyl 4-hydroxy cinnamate | 0.4600 |
| 4 | 2497 | 3-butenyldesulfoglucosinolate | 0.3840 |
| 5 | 4415 | 3β-acetoxyolean-18-en-28-oic acid | 0.3610 |
| 6 | 2853 | Butiin | 0.2890 |
| 7 | 1018 | 5-Carboxypyranocyanidin 3-O-(6''-O-malonyl-beta-glucopyranoside) | 0.2780 |
| 8 | 2507 | (+)-N-(methoxycarbonyl)-N-norboldine | 0.2770 |
| 9 | 603 | Gibberellin A12 | 0.2700 |
| 10 | 4332 | (9R,10S)-dihydroxyoctadecanoic acid | 0.2280 |
| 11 | 2253 | Δ6-protoilludene | 0.2220 |
| 12 | 1426 | oleocanthal | 0.1670 |
| 13 | 2355 | S-[(E)-Prop-1-enyl]-L-cysteine S-oxide | 0.1540 |
| 14 | 2673 | (−)-homalomenol D | 0.1510 |
| 15 | 4534 | erythrodiol | 0.1420 |
| Class 11 - Muscle and Bone | | | |
| 1 | 4078 | 14-deoxo-3-O-propionyl-5,15-di-O-acetyl-7-O-benzoylmyrsinol-14beta-nicotinoate | 0.1870 |
| 2 | 1804 | Euphorbiaproliferin I | 0.1250 |
| 3 | 4570 | Euphorbiaproliferin G | 0.1070 |
| 4 | 2591 | Proliferin A | 0.0973 |
| 5 | 285 | Euphorbiaproliferin A, (rel)- | 0.0852 |
| 6 | 2504 | 14-deoxo-3-O-propionyl-5,15-di-O-acetyl-7-O-benzoylmyrsinol-14beta-acetate | 0.0810 |
| 7 | 858 | Euphorbiaproliferin E | 0.0748 |
| 8 | 2980 | glucoiberverin(1−) | 0.0696 |
| 9 | 1513 | Euphorbiaproliferin J | 0.0666 |
| 10 | 2146 | Euphorbiaproliferin D | 0.0641 |
| 11 | 44 | Euphorbiaproliferin H | 0.0569 |
| 12 | 4589 | (−)-(12E,2S,3S,4R,5R,6R,9S,11S,15R)-3-acetoxy-15-benzoyloxy-5,6-epoxylathyr-12- | 0.0562 |
| 13 | 250 | Euphorprolitherin C | 0.0556 |
| 14 | 811 | Euphorbiaproliferin F | 0.0290 |
| 15 | 3435 | homostachydrine | 0.0175 |
| Class 13 - The Nervous System | | | |
| 1 | 131 | isoamylol | 0.2240 |
| 2 | 4335 | rubiarboside G 28-acetate | 0.1690 |
| 3 | 434 | pterostilbene | 0.1290 |
| 4 | 4114 | fertaric acid | 0.0831 |
| 5 | 2065 | ,3,6-trihydroxy-2-methyl-9,10-anthraquinone-3-O-α-L-rhamnopyranosyl-(1→2)-β-D-glucopyranoside | 0.0494 |
| 6 | 75 | Isoterchebin; Trapain | 0.0396 |
| 7 | 49 | methyl tert-butyl ether | 0.0356 |
| 8 | 1610 | cyanidin 3-O-(6-O-acetyl-β-D-glucoside) | 0.0314 |
| Class 15 - Respiratory Diseases | | | |
| 1 | 1197 | 2,5-Dihydro-5-oxofuran-2-acetate; 2-Oxo-2,5-dihydrofuran-5-acetate; Muconolactone; 5-Oxo-2,5-dihydrofuran-2-acetate; 4-Carboxymethyl-4-hydroxyisocrotonolactone | 0.4300 |
| 2 | 1875 | 2-Butenoate; 2-Butenoic acid; Crotonic acid; 3-Methylacrylic acid | 0.2730 |
| 3 | 4624 | 6-epi-guttiferone J | 0.2250 |
| 4 | 531 | Isoacolamone | 0.1390 |
| 5 | 3846 | Apo-[3-methylcrotonoyl-CoA:carbon-dioxide ligase (ADP-forming)]; Apo-[methylcrotonoyl-CoA-carboxylase] | 0.1320 |
| 6 | 4097 | kaempferol 3-O-α-L-arabinopyranosyl-7-O-α-L-rhamnopyranoside | 0.1200 |
| 7 | 2264 | cis-Methyl isoeugenol | 0.0985 |
| 8 | 848 | 2(3H)-Furanone; alpha-Crotonolactone | 0.0858 |
| 9 | 196 | (E)-4-(Trimethylammonio)but-2-enoate; Crotono-betaine; Crotonobetaine | 0.0706 |
| 10 | 96 | Calacone | 0.0677 |
| 11 | 1534 | Crotonoyl-CoA; Crotonyl-CoA; 2-Butenoyl-CoA; trans-But-2-enoyl-CoA; But-2-enoyl-CoA; (E)-But-2-enoyl-CoA | 0.0477 |
| 12 | 4675 | Isoshyobunone | 0.0400 |
| 13 | 2133 | 2-(3,4-dihydroxyphenyl)-ethyl-O-β-D-glucopyranoside | 0.0395 |
| 14 | 3706 | 32-hydroxy-ent-guttiferone M | 0.0238 |
| 15 | 312 | kaempferol 3-O-β-D-galactopyranosyl-7-O-α-L-rhamnopyranoside | 0.0208 |
| Class 16 - Skin and Connective Tissue | | | |
| 1 | 1306 | 5-Hydroxyindoleacetate | 0.4880 |
| 2 | 2846 | Taxifolin 3'-glucoside | 0.4520 |
| 3 | 3586 | Oleandomycin 2'-O-phosphate | 0.2960 |
| 4 | 4306 | Oleanolic acid; 3beta-Hydroxyolean-12-en-28-oic acid; Astrantiagenin C; Caryophyllin; Oleanolate | 0.2560 |
| 5 | 3624 | (+)-alpha-Atlantone | 0.2220 |
| 6 | 1966 | (+)-Wikstromol | 0.2090 |
| 7 | 4616 | 5-Hydroxyindoleacetaldehyde | 0.2070 |
| 8 | 1970 | Oleandrin | 0.1900 |
| 9 | 4201 | Cedrinoside | 0.1680 |
| 10 | 438 | Indole-3-acetaldehyde; 2-(Indol-3-yl)acetaldehyde; Indoleacetaldehyde | 0.1600 |
| 11 | 3528 | 5-Hydroxyindoleacetylglycine | 0.1390 |
| 12 | 2461 | Himaphenolone | 0.1360 |
| 13 | 2316 | Coniferyl aldehyde; Coniferaldehyde; 4-Hydroxy-3-methoxycinnamaldehyde; Ferulaldehyde | 0.1320 |
| 14 | 1839 | 11alpha-Hydroxy-beta-amyrin; 11alpha-Dihydroxyolean-12-ene | 0.1020 |
| 15 | 2866 | Cedrin | 0.1010 |
| Class 17 - The Urinary System | | | |
| 1 | 908 | Glyoxylic acid | 0.3450 |
| 2 | 2438 | Resokaempferol | 0.2800 |
| 3 | 322 | Biochanin A | 0.2750 |
| 4 | 3581 | Dihydrozeatin | 0.2540 |
| 5 | 2549 | Homoferreirin | 0.2480 |
| 6 | 3752 | Pyruvic acid | 0.2010 |
| 7 | 1589 | graphite | 0.1970 |
| 8 | 1526 | Oxalic acid | 0.1840 |
| 9 | 97 | Kaempferol 3-apiosyl-(1->2)-glucoside | 0.1840 |
| 10 | 4026 | Soyasaponin I | 0.1630 |
| 11 | 1067 | 2-(methyldithio)pyridine-N-oxide | 0.1520 |
| 12 | 2898 | Liquiritigenin | 0.1490 |
| 13 | 3934 | Garbanzol | 0.1270 |
| 14 | 1266 | Medicagol | 0.1200 |
| 15 | 4478 | Biochanin A 7-O-glucoside | 0.1110 |
